# Transcriptomic and protein analysis of human cortex reveals genes and pathways linked to NPTX2 disruption in Alzheimer’s disease

**DOI:** 10.1101/2025.10.17.683150

**Published:** 2025-10-20

**Authors:** Yuelin Lao, Mei-Fang Xiao, Shiyu Ji, Ignazio S. Piras, Anna Bonfitto, Serena Song, Arailym Aldabergenova, Chan-Hyun Na, Jennifer Sloan, Adriel Trejo, Changiz Geula, Emily J. Rogalski, Claudia H. Kawas, Maria M. Corrada, Geidy E. Serrano, Thomas G. Beach, Juan C. Troncoso, Matthew J. Huentelman, Carol A. Barnes, Paul F. Worley, Carlo Colantuoni

**Affiliations:** 1 Solomon H. Snyder Department of Neuroscience, Johns Hopkins University School of Medicine, Baltimore, MD; 2 Translational Genomics Research Institute, Phoenix, AZ; 3 Department of Neurology, Institute for Cell Engineering, Johns Hopkins University School of Medicine, Baltimore, MD; 4 Mesulam Center for Cognitive Neurology & Alzheimer’s Disease, Northwestern University School of Medicine, Chicago, IL; 5 Healthy Aging & Alzheimer’s Research Care (HAARC) Center, University of Chicago, Chicago, IL; 6 University of California, Irvine, Irvine, CA; 7 Banner Sun Health Research Institute, Sun City, AZ; 8 Department of Pathology, Johns Hopkins University School of Medicine, Baltimore, USA; 9 Evelyn F. McKnight Brain Institute, University of Arizona, Tucson, AZ; 10 Department of Neurology, Johns Hopkins University School of Medicine, Baltimore, MD; 11 Institute for Genome Sciences, University of Maryland, Baltimore, USA

## Abstract

The expression of NPTX2, a neuronal immediate early gene (IEG) essential for excitatory–inhibitory balance, is altered in the earliest stages of cognitive decline that anticipate Alzheimer’s disease (AD). Here, we use NPTX2 as a point of reference for Omics studies to identify genes and pathways linked to its position in AD onset and progression. We integrated bulk RNA sequencing from 575 middle temporal gyrus (MTG) samples across four cohorts together with targeted proteomics in the same samples using parallel reaction monitoring–mass spectrometry in 135 representative cases, focusing on 20 curated proteins spanning synaptic, trafficking, lysosomal, and regulatory categories. NPTX2 RNA and protein were significantly reduced in AD, and to a lesser extent in mild cognitive impairment (MCI) samples. BDNF, VGF, SST, and SCG2 correlated with both NPTX2 mRNA and protein. We identified NPTX2 correlated synaptic and mitochondrial programs that were negatively correlated with lysosomal and chromatin/stress modules. Gene set enrichment analysis (GSEA) of NPTX2 correlations across all samples confirmed broad alignment with synaptic and mitochondrial compartments, while more NPTX2-specific associations were observed with proteostasis and translation regulator pathways, which were weakened in AD. In contrast, correlation of NPTX2 protein with transcriptomic profiles revealed negative associations with stress-linked transcription regulator RNAs (FOXJ1, ZHX3, SMAD5, JDP2, ZIC4), which were strengthened in AD. Studies position NPTX2 as a hub of an activity-regulated “plasticity cluster” (BDNF, VGF, SST, SCG2) that encompasses interneuron function and is embedded on a neuronal/mitochondrial integrity axis that is inversely coupled to lysosomal/chromatin–stress programs. In AD, these transcript-level correlations broadly weaken, and stress-linked transcriptional regulators become more prominent, suggesting a role in NPTX2 loss of function.

## INTRODUCTION

NPTX2 is an immediate early gene (IEG) and synaptic protein that plays a key role in memory consolidation by regulating the balance of excitation and inhibition within neuronal ensembles [1, 2]. In Alzheimer’s disease (AD), NPTX2 mRNA and protein levels are reduced in the postmortem neocortex compared to cognitively normal, age-matched controls [3]. Notably, this reduction is not observed in individuals who exhibit AD pathology but remained cognitively intact until death—commonly referred to as “asymptomatic AD” [4] or “high pathology controls” [5, 6]—suggesting that NPTX2 expression may represent a resilience factor for preserved cognition. This hypothesis is further supported by glycoproteomic screens identifying NPTX2 as a top candidate linked to cognitive resilience in older adults [7]. In cerebrospinal fluid (CSF), NPTX2 levels are decreased in individuals with mild cognitive impairment (MCI) and AD [8–10], and its inclusion improves the diagnostic performance of established AD biomarkers, such as the Aβ42/40 ratio and tau proteins [3, 11]. Longitudinal studies show that NPTX2 levels, in combination with tau measures, can predict time to onset of cognitive symptoms [12]. Moreover, in large-scale, unbiased CSF biomarker screens, the ratio of YWHAZ/NPTX2 has emerged as a highly effective diagnostic indicator [13].

The functional “life cycle” of NPTX2 involves multiple cell biological processes. Its dynamic, activity-dependent transcription and translation follow canonical IEG mechanisms [1, 14]. However, as a glycosylated presynaptic protein, NPTX2 also requires proper processing in the Golgi, assembly into disulfide-linked complexes with NPTX1 and NPTXR, fast axonal transport to presynaptic terminals, activity-dependent release, and subsequent shedding during sleep-related circuit refinement [15–17]. Among the NPTX family members, NPTX2 is uniquely IEG-regulated and is essential for targeting the NPTX complex to excitatory synapses on Parvalbumin (PV) interneurons [3, 15]. At these sites, the NPTX1/2/R complex strengthens excitatory input by binding postsynaptic AMPA receptors, particularly GluA4 [2, 18–20]. This action contributes to maintaining the excitation-inhibition balance within sparse, behaviorally engaged neuronal assemblies [2] and transgene expression rescues altered E/I balance in aged [21] and Aß amyloid models of AD [22]. Additionally, synaptically localized NPTX2 inhibits complement factor C1q, which upon shedding becomes active and can trigger microglia-mediated synapse elimination, a mechanism potentially relevant to both physiological circuit refinement and pathological synapse loss in AD [23]. Thus, NPTX2 function relies on multiple facets of cell biology—including transcriptional regulation, glycoprotein processing, synaptic targeting, and immune modulation—that may be disrupted in neurodegenerative disease. Studying its expression and function could therefore provide insight into early, cell-type specific mechanisms of AD pathogenesis and resilience.

In the present study, we examine NPTX2 mRNA and protein as distinct indicators of NPTX2 expression and function in the human brain, with the goal of expanding our understanding of the molecular causes and consequences of NPTX2 loss. Using unbiased informatic analyses, we assess co-regulated genes and pathways associated with both RNA and protein-level changes in NPTX2. Our proteomics panel includes general synaptic markers and proteins implicated in neuronal functions relevant to AD, such as vesicular trafficking, autophagy-lysosomal pathways, and mitochondrial metabolism. We find strong associations of NPTX2 expression with growth factors such as BDNF and VGF, as well as selective enrichment of pathways related to transcriptional regulation, translation, protein folding, and mitochondrial function. These findings expand our current understanding of NPTX2 biology and offer new mechanistic links between NPTX2 loss of function and early pathological changes in AD.

## RESULTS

### Study Design and Cohort Overview

**Figure 1.**
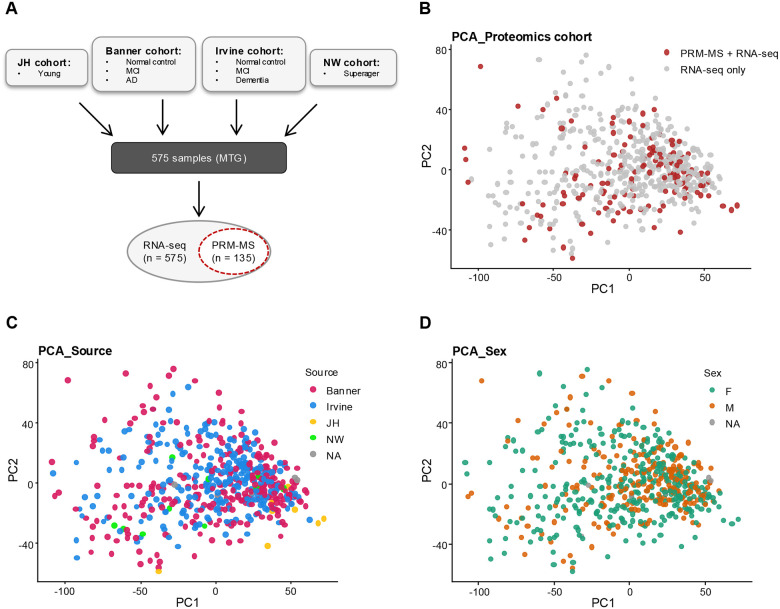
Study Design and Cohort Overview. (A) Schematic of four independent postmortem brain cohorts included in the study. Johns Hopkins contributed young controls; Banner contributed controls, mild cognitive impairment (MCI), and Alzheimer’s disease (AD) cases; Irvine contributed controls, MCI, and dementia cases; Northwestern contributed superagers, grouped with controls. After quality control, 575 samples were retained for bulk RNA-seq, of which 135 representative cases were selected for targeted proteomic analysis by PRM-MS. MTG: middle temporal gyrus. (B) Proteomics cohort. 135 brains that also have mass-spectrometry data are shown in red, which are inter-mixed with the full cohort across principal component (PC) space, indicating that the proteomics subset spans the transcriptomic diversity of the study and is not confined to a particular cluster or outlier region. (C) Source. Brains are colored by tissue source: Banner, Irvine, Johns Hopkins (JH), Northwestern (NW), or NA when unannotated. (D) Sex. Female (green), male (orange), and NA (grey).

We analyzed postmortem middle temporal gyrus (MTG) tissue from four independent cohorts (Fig. 1). The Johns Hopkins cohort provided young controls, the Banner cohort contributed cognitively normal controls, mild cognitive impairment (MCI), and Alzheimer’s disease (AD) dementia cases, the Irvine cohort included controls, MCI, and dementia cases, and the Northwestern cohort contributed superagers, which were grouped within the control category. Following quality control, bulk RNA-seq data were available for 575 samples across these diagnostic groups.

A subset of 135 representative samples was selected for proteomic analysis using parallel reaction monitoring–mass spectrometry (PRM-MS), which were dispersed throughout the principal component (PC) score space of the 575-sample bulk RNA-seq dataset and did not form a tight cluster, supporting that the subset is representative of the full cohort. The proteomics panel targeted 20 genes spanning synaptic, trafficking, lysosomal, and regulatory categories (see methods). PRM-MS was chosen for its high sensitivity and reproducibility in quantifying low-abundance synaptic proteins, enabling robust detection of NPTX2 and related candidates. This multi-omics design provided complementary resolution: RNA-seq offered a broad, transcriptome-wide survey, while PRM-MS enabled more direct assessment of functional protein-level relationships for NPTX2 and selected partners.

### NPTX2 RNA and Protein Expression Across Diagnostic Groups

**Figure 2.**
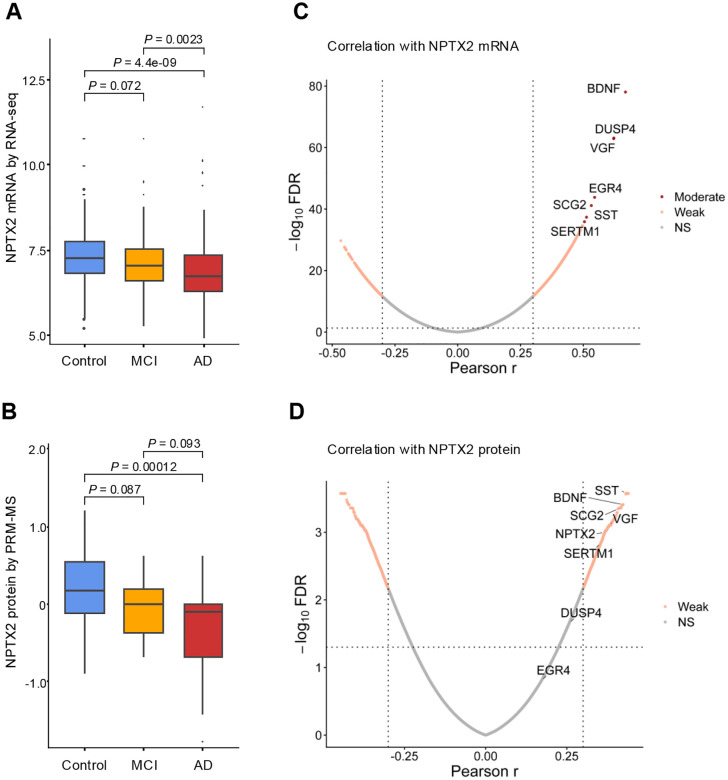
NPTX2 expression and correlation structure. (A) Boxplots of NPTX2 mRNA expression across Control, MCI, and AD groups. Control, n = 180; MCI, n = 83; AD, n = 218. (B) Boxplots of NPTX2 protein abundance measured by PRM-MS across the same diagnostic groups. Control, n = 57; MCI, n = 20; AD, n = 38. Boxplots display the median (horizontal line), interquartile range (box), and whiskers (1.5 × IQR). (C) Volcano plot of Pearson correlations between all RNA transcripts (x-axis: correlation coefficient r) and NPTX2 mRNA expression (y-axis: −log_10_FDR). Vertical dotted lines mark correlation thresholds (|r| = 0.30), and the horizontal dotted line marks the FDR = 0.05 significance threshold. Genes exceeding both correlation and FDR cutoffs are colored, with the eight transcripts having |r| > 0.5 labeled. (D) Volcano plot of correlations between RNA transcripts and NPTX2 protein levels, plotted as in panel C.

NPTX2 expression was assessed across Control, MCI, and AD groups at both the RNA and protein levels. Bulk RNA-seq analysis showed a stepwise reduction in expression (Control > MCI> AD; Fig. 2A). Pairwise Wilcoxon rank-sum tests with false discovery rate correction confirmed significant differences for two comparisons: Control versus AD (*p* ≤ 0.0001), and MCI versus AD (*p* ≤ 0.01). Proteomic analysis by PRM-MS showed a parallel decreasing trend (Fig. 2B). Significant differences were observed between Control and AD (*p* ≤ 0.001). In contrast, the comparison between MCI and AD did not reach significance.

The observed reductions of NPTX2 RNA and protein across diagnostic groups are consistent with prior reports showing profound decreases of NPTX2 in the postmortem cortex of AD patients [3]. Our data extends these findings by demonstrating a graded decline, with reductions already evident at the MCI stage (NPTX2 RNA: *p* ≈ 0.07, NPTX2 protein: *p* ≈ 0.09) but more significant at AD stage (NPTX2 RNA: *p* ≤ 0.001, NPTX2 protein: ≈ 0.001).

### Transcript–Protein Correspondence of NPTX2

To identify transcripts that correlate with NPTX2 at both the RNA and protein levels, we calculated gene-wise Pearson correlation coefficients with NPTX2 using bulk RNA-seq and PRM-MS data. The RNA-based volcano plot showed a right-skewed distribution, indicating a predominance of positive correlations with NPTX2 expression (Fig. 2C). In contrast, the proteomic volcano plot displayed a more symmetric distribution, with both positive and negative correlations represented (Fig. 2D). NPTX2 showed self-correlation in both modalities, with RNA–protein correlation in the moderate range (r ≈ 0.37, FDR < 0.05).

In the RNA volcano plot, eight genes showed strong positive correlation with NPTX2 expression (overall r > 0.5; Fig. 2C). These included NPTX2, BDNF, DUSP4, EGR4, VGF, SCG2, SST, and SERTM1. RNA correlation with NPTX2 protein was preserved for BDNF, VGF, SCG2, SST, and SERTM1, each showing moderate RNA–protein associations (r ≈ 0.35–0.43, FDR < 0.05; Fig. 2D). In contrast, DUSP4 (r ≈ 0.26, FDR ≈ 0.02) and EGR4 (r ≈ 0.17, FDR < 0.05) did not reach significance in the proteomic analysis, indicating reduced transcript–protein correspondence. Gene ontology information for these NPTX2-associated transcripts is summarized in supplemental table 1.

### Top RNA Correlates of NPTX2 and Their Protein-Level Associations

**Figure 3.**
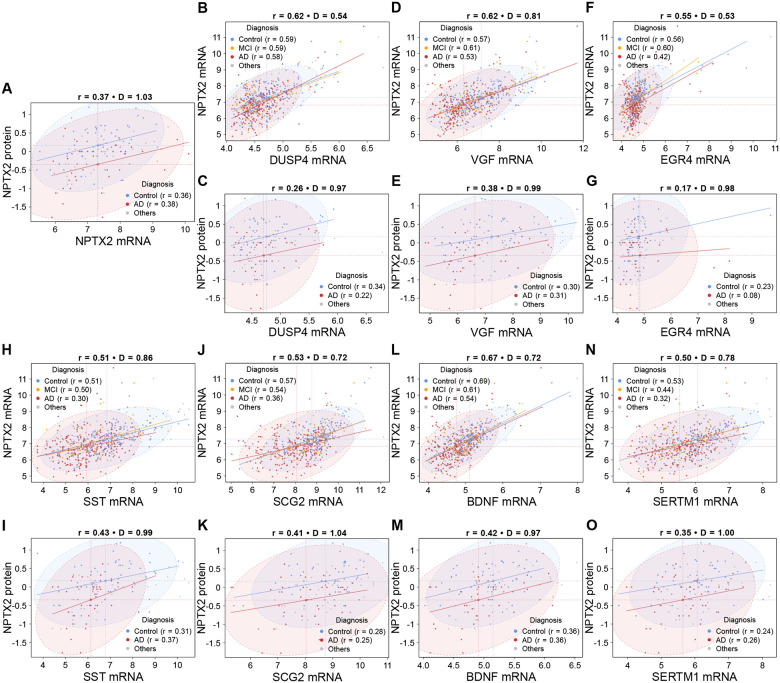
Gene-specific scatterplots of RNA–protein correspondence. (A–O) Scatterplots showing gene-level RNA–RNA correlations (B, D, F, H, J, L, and N) and RNA–protein correlations (A, C, E, G, I, K, M, and O) for NPTX2. Top seven RNA correlates (DUSP4, VGF, EGR4, SST, SCG2, BDNF, SERTM1) are shown here. Each point represents one sample, colored by diagnostic group (Control = blue, MCI = orange, AD = brown). Ordinary least-squares regression lines are fitted for each group (Control, MCI, AD). Two vertical dashed lines indicate the group mean gene expression (x-axis) for Control and AD; two horizontal dashed lines indicate the group mean NPTX2 expression (y-axis) for Control and AD. For Control and AD, 95% confidence ellipses are drawn around the data points, showing the distribution of each group. Panel titles report the overall correlation coefficient across all samples and the Mahalanobis distance (D) between Control and AD group centroids.

We next examined gene-level associations with NPTX2 within diagnostic groups (Control, MCI, AD); groups with insufficient sample size (Young control, non-AD dementia, and other pathology case groups in RNA-seq dataset and young control, non-AD dementia, other pathology, and MCI case groups in PRM-MS dataset) were omitted. Across all samples, NPTX2 RNA and protein were moderately correlated (Fig. 3A). In the NPTX2 RNA–MS panel, the within-group RNA–protein correlation changed little (Control r = 0.36; AD r = 0.38), whereas the Mahalanobis distance reached D = 1.03, indicating strong separation driven by a downward shift of NPTX2 protein in AD. Along the x-axis, NPTX2 RNA was comparatively unchanged, so for this anchor panel the Control centroid sits higher than AD on both axes.

To test whether other gene–NPTX2 relationships also shifted within groups, we focused on the seven transcripts most strongly correlated with NPTX2 RNA (r > 0.5 in Fig. 2C). Using Pearson correlation, we asked whether these gene–NPTX2 associations were maintained, weakened, or strengthened in AD compared to Control. Because RNA abundance does not always translate to protein abundance [24], we evaluated both the *transcript layer* (gene RNA vs NPTX2 RNA) and the *protein layer* (gene RNA vs NPTX2 protein).

Of these seven transcripts, five retained significant association with NPTX2 protein (Fig. 3). BDNF decorrelated at the transcript layer (r = 0.69 → 0.54), while the RNA–protein correlation remained stable at 0.36 in both groups; separation increased from D = 0.72 in RNA to D = 0.97 in RNA–MS, moving from moderate to moderate-to-strong and consistent with lower BDNF RNA together with lower NPTX2 protein in AD (Fig. 3L and 3M). VGF showed mild transcript-level decorrelation (0.57 → 0.53) with a marginal rise in RNA–protein correlation (0.30 → 0.31); the separation increased from D = 0.81 to D = 0.99, again moderate to moderate-to-strong, indicating coordinated reductions of VGF RNA and NPTX2 protein in AD (Fig. 3D and 3E). SCG2 displayed a broader weakening across layers: transcript correlation decorrelated from 0.57 to 0.36 and RNA–protein correlation fell from 0.28 to 0.25; separation rose from D = 0.72 to D = 1.04, reflecting strong group separation and joint reductions of SCG2 RNA and NPTX2 protein in AD (Fig. 3J and 3K). SST decorrelated at the transcript layer (0.51 → 0.30) while over-correlating modestly at the RNA–protein layer (0.31 → 0.37); separation increased from D = 0.86 to D = 0.99, moderate to moderate-to-strong, indicating lower SST RNA together with lower NPTX2 protein in AD (Fig. 3H and 3I). SERTM1 likewise decorrelated at the transcript layer (0.53 → 0.32) with slight RNA–protein over-correlating (0.24 → 0.26); separation rose from D = 0.78 to D = 1.00, moderate to strong, consistent with concurrent reductions of SERTM1 RNA and NPTX2 protein in AD (Fig. 3N and 3O).

By contrast, the immediate-early transcripts showed limited correspondence at the protein layer. DUSP4 maintained a stable transcript correlation with NPTX2 (0.59 vs. 0.58) but decorrelated at the RNA–protein layer (0.34 → 0.22); separation increased from D = 0.54 to D = 0.97, moving from little to moderate-to-strong, indicating joint reductions of DUSP4 RNA and NPTX2 protein in AD (Fig. 3B and 3C). EGR4 decorrelated at both layers (transcript 0.56 → 0.42; RNA–protein 0.23 → 0.08); separation increased from D = 0.53 to D = 0.98, little to moderate-to-strong. In RNA–MS space this pattern indicates lower EGR4 RNA together with lower NPTX2 protein in AD; in RNA–RNA space the separation is dominated by lower NPTX2 RNA in AD while EGR4 RNA largely overlaps between groups (Fig. 3F and 3G). Collectively, these results show pervasive transcript-level decorrelation among top NPTX2 correlates in AD, with gene-specific behavior at the protein layer: BDNF and VGF maintained their correlations with NPTX2 across groups at both layers; SST and SERTM1 display modest over-correlating; SCG2 weakens across layers; and DUSP4/EGR4 lose transcript– NPTX2 protein correspondence.

### Correlation Heatmap and Module Structure

**Figure 4.**
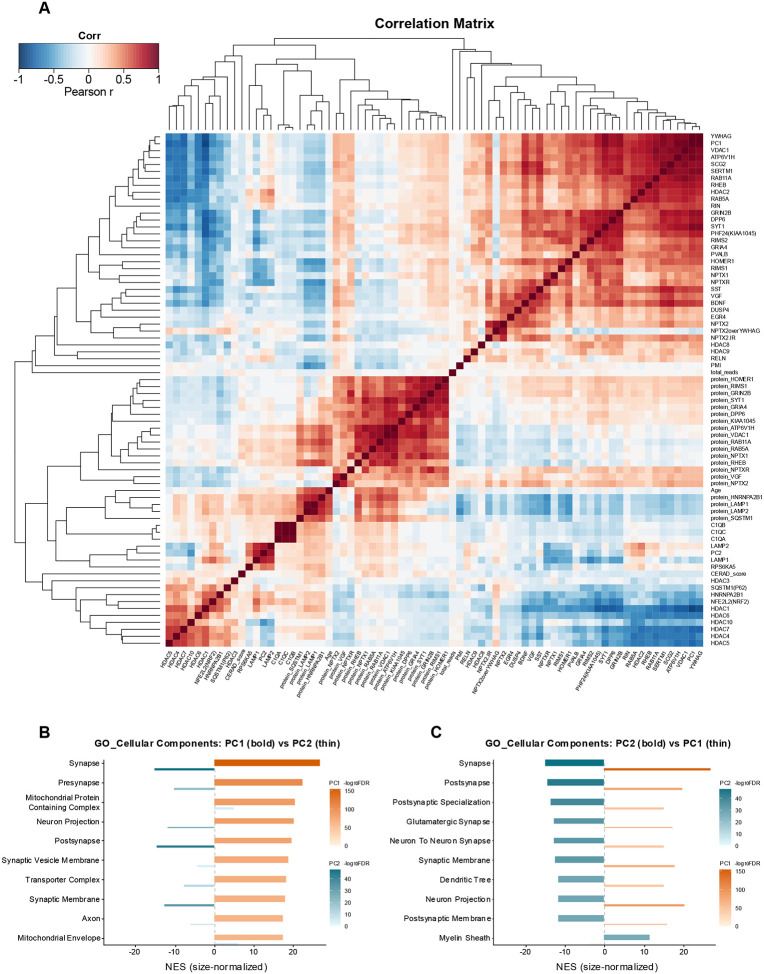
Correlation structure and component-wise enrichment analysis. (A) Pearson correlation heatmap across selected RNA transcripts, proteomic variables, and metadata features. Variables are hierarchically clustered (average linkage, distance = 1 – r). Color intensity denotes correlation strength (blue = negative, red = positive). Distinct modules corresponding to synaptic machinery, mitochondrial and vesicular transport, lysosomal–autophagic factors, chromatin/stress regulators, activity-dependent transcripts, and Alzheimer’s pathology metrics are evident. (B) Top Gene Ontology Cellular Component (GO:CC) terms enriched among PC1-associated genes or proteins, ranked by absolute normalized enrichment score (NES). Bold bars represent NES for PC1, and thin bars directly beneath indicate NES for PC2. Bar color encodes −log_10_FDR, reflecting enrichment significance. (C) Analogous to (B), but ranked by absolute PC2 NES, with bold bars denoting PC2 and thin bars showing PC1. Bar color likewise represents −log_10_FDR.

We next constructed a correlation matrix spanning RNA transcripts of genes that are known or could be functionally linked to NPTX2 (see Methods), targeted proteomic variables, and metadata features, and clustered the resulting Pearson correlation coefficients by hierarchical average linkage (Fig. 4A), along with Principal Component 1 (PC1) and Principal Component 2 (PC2), derived from a principal component analysis (PCA) of the 575-sample RNA expression matrix. The heatmap revealed distinct RNA and proteomic modules with strong internal coherence. On the RNA side, five major branches were identified. Module 1/1/1, a mitochondrial/vesicle-transport cluster, contained YWHAG, PC1, VDAC1, ATP6V1H, SCG2, SERTM1, RAB11A, RHEB, HDAC2, RAB5A, and RIN. Module 1/1/2 comprised canonical synaptic machinery and postsynaptic scaffolds, including GRIN2B, DPP6, SYT1, PHF24, RIMS2, GRIA4, PVALB, HOMER1, RIMS1, NPTX1, and NPTXR. Module 1/2/1 was centered on NPTX2 and grouped activity-dependent transcripts (SST, VGF, BDNF, DUSP4, EGR4), forming a tight plasticity-associated block. A smaller branch, Module 1/3/1, combined chromatin regulators HDAC8 and HDAC9 with the extracellular matrix protein RELN. Finally, Module 1/4/1 contained technical variables, including post-mortem interval (PMI) and sequencing depth; PMI was negatively correlated with protein LAMP1, SQSTM1, and HNRNPA2B1.

Incorporating proteomic data yielded six additional clusters. Module 2/1/1 grouped synaptic proteins (protein HOMER1, RIMS1, GRIN2B, SYT1, GRIA4, DPP6, PHF24). Module 2/1/2 comprised vesicle and Rab GTPases (protein ATP6V1H, VDAC1, RAB11A, RAB5A, NPTX1, RHEB, NPTXR), while Module 2/1/3 contained VGF and NPTX2. Module 2/2/1 grouped age and lysosomal/clearance features (age continuous, protein HNRNPA2B1, LAMP1, LAMP2, SQSTM1). Module 2/2/2 contained complement factors (C1QA, C1QB, C1QC), and Module 2/2/3 combined glial/inflammatory signals (LAMP2, PC2, LAMP1, RPS6KA5). Module 2/3/1 represented a chromatin/stress/pathology cluster (CERAD score, protein HDAC3, SQSTM1, HNRNP2B1, NFE2L2, and HDAC1, 4–10). Cross-modality contrasts indicated weak positive alignment between RNA neuronal modules and MS synaptic clusters 2/1/1 and 2/1/3 (protein NPTX2, VGF), whereas the trafficking/outer-membrane cluster 2/1/2 was more frequently anti-correlated with RNA synaptic modules.

At the systems level, neuronal/synaptic programs (Modules 1/1/1, 1/1/2, 1/2/1 on the RNA side and 2/1/1, 2/1/3 on the MS side) were inversely correlated with stress- and pathology-related programs (Modules 2/2/1, 2/2/3, 2/3/1), while the complement cluster (2/2/2) showed a weaker and less uniform pattern than lysosomal and chromatin/stress modules. Principal component analysis of the RNA matrix supported these relationships: PC1 aligned with the mitochondrial/vesicle-transport branch (Module 1/1/1), capturing a global neuronal integrity signature (Fig. 4B), whereas PC2 clustered with lysosomal and glial features and showed negative enrichment for postsynaptic/dendritic structures and positive enrichment for myelin-associated transcripts (Fig. 4C).

### Cellular Component Enrichment of NPTX2-Correlated Genes (RNA and Protein)

**Figure 5.**
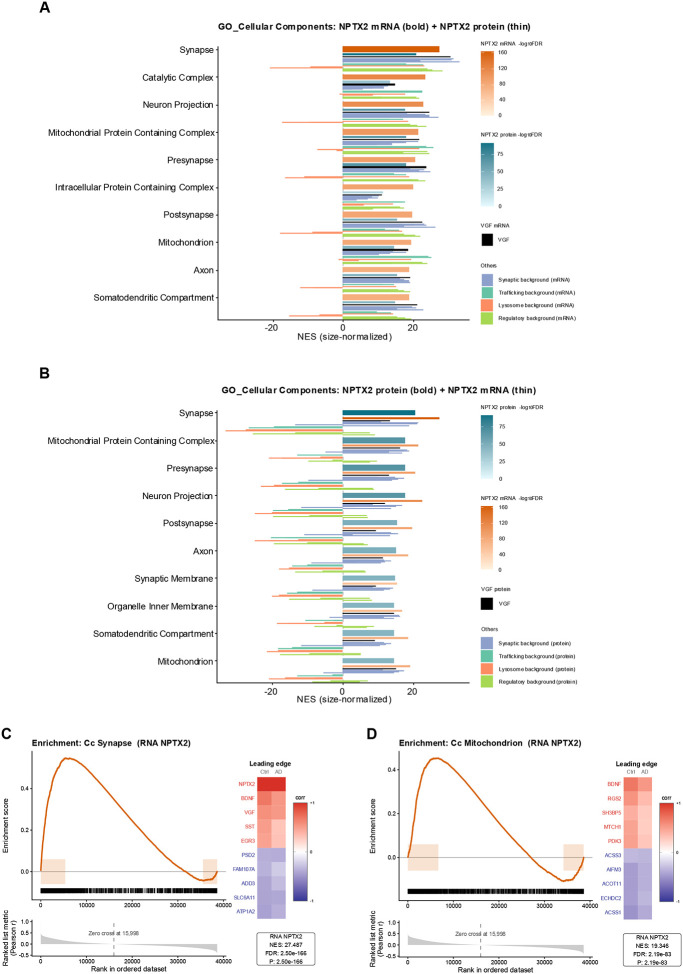
Gene set enrichment of NPTX2-associated cellular components. (A) Barplot of the top Gene Ontology Cellular Component (GO:CC) terms enriched among genes correlated with NPTX2 mRNA. Bold bars indicate normalized enrichment scores (NES) for NPTX2 mRNA, with thinner bars below showing cross-modality NES for NPTX2 protein. Additional narrow background bars represent per-gene NES values for curated comparator genes in the same modality as the bold bar. Background genes are color-coded by functional category (synaptic, trafficking, lysosomal, regulatory), with VGF highlighted separately. Bar color encodes −log_10_FDR, reflecting enrichment significance. Background values were computed using the same enrichment method as for NPTX2, providing direct reference points for assessing the magnitude and specificity of NPTX2 enrichment within synaptic and metabolic networks. (B) Barplot of the top GO:CC terms enriched among genes correlated with NPTX2 protein, displayed as in (A) with bold bar = NPTX2 protein and thin bar = NPTX2 mRNA. (C) Enrichment plot for GO:CC Synapse, showing the ranked gene list (x-axis), vertical ticks marking set members, and the running-sum enrichment score (ES). The NES and leading-edge contributors are annotated. (D) Enrichment plot for GO:CC Mitochondrion, displayed as in (C).

To evaluate pathway-level associations with NPTX2, we applied gene set enrichment analysis (GSEA) and reported results as normalized enrichment scores (NES) [25]. NES rescales the raw enrichment statistic to account for gene set size and variation across permutations, providing a standardized measure of whether genes in each pathway are disproportionately correlated with NPTX2 compared to chance. This makes NES directly comparable across pathways and between RNA- and protein-based datasets. Across both RNA and protein correlation profiles, the top Cellular Component enrichments prominently featured neuronal compartments, including synapse, neuron projection, mitochondrion, and axon (Fig. 5A and 5B). Synapse and mitochondrion were consistently observed but not uniquely enriched, as NES values for NPTX2 were matched or exceeded by several curated background genes (see methods), which we define as reference synaptic, trafficking, lysosomal, and regulatory markers measured in both modalities. Other terms such as catalytic complex, somatodendritic compartment, organelle inner membrane, and mitochondrial protein-containing complex also ranked highly but lacked specificity, due to comparable enrichment by trafficking or regulatory background genes. Redundant cellular component terms — including presynapse, postsynapse, synaptic membrane, and axon — were excluded from further interpretation because their corresponding parent categories (e.g., Synapse, Neuron projection) were already represented.

The enrichment curve for the synapse term (Fig. 5C) showed a sharp left-skew with high NES (27.487). The five most positive contributors were NPTX2, BDNF, VGF, SST, and EGR3, and the five most negative were ATP1A2, SLC6A11, ADD3, FAM107A, and PSD2. In controls, correlations were strong across both positive and negative contributors, whereas in AD the structure was weakened.

The mitochondrion category (Fig. 5D) showed enrichment with NES = 19.345. The five most positive contributors were BDNF, RGS2, SH3BP5, MTCH1, and PDK3, while the five most negative were ACSS1, ECHDC2, ACOT11, AIFM3, and ACSS3. In controls, correlations between NPTX2 and mitochondrial genes were stronger than in AD, where the associations diminished.

### Molecular Function Enrichment of NPTX2-Correlated Genes (RNA and Protein)

**Figure 6.**
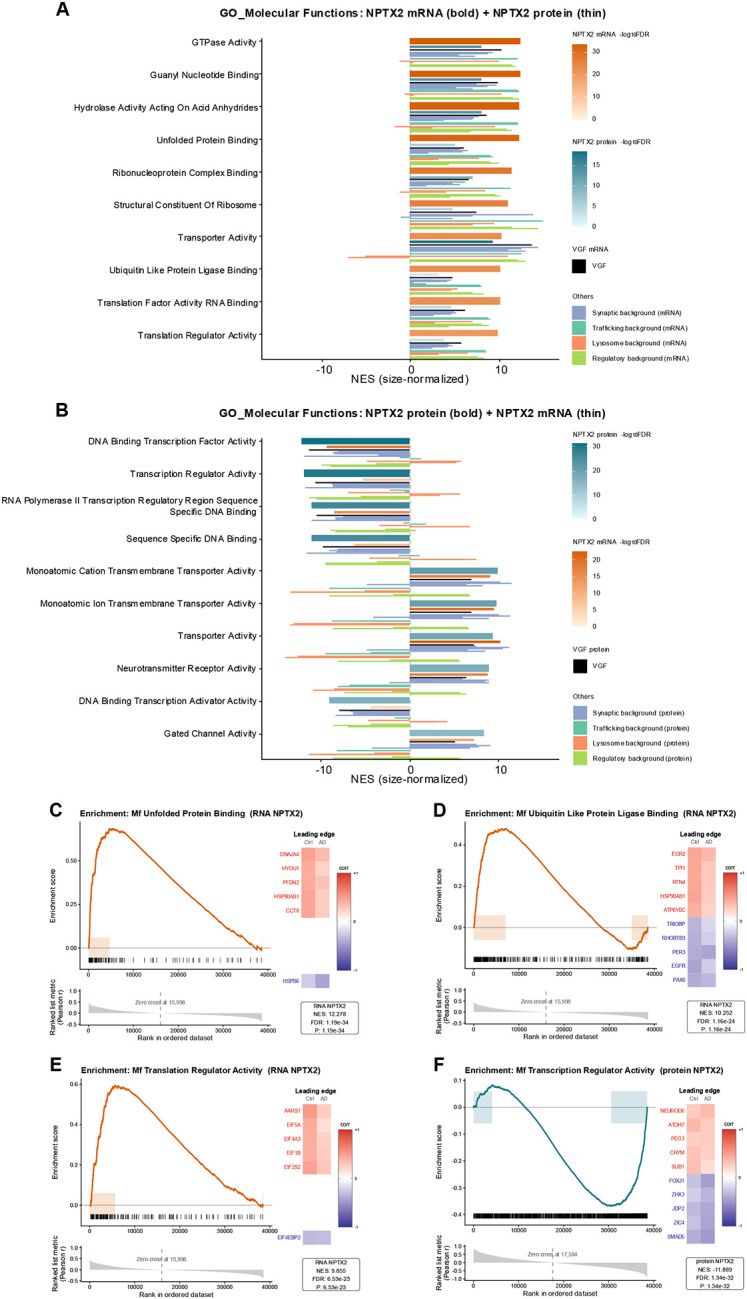
Gene set enrichment of NPTX2-associated molecular functions. (A) Barplot of the top Gene Ontology Molecular Function (GO:MF) terms enriched among genes correlated with NPTX2 mRNA. Bold bars indicate normalized enrichment scores (NES) for NPTX2 mRNA, with thinner bars below showing cross-modality NES for NPTX2 protein. Additional narrow background bars represent per-gene NES values for curated comparator genes in the same modality as the bold bar. Background genes are color-coded by functional category (synaptic, trafficking, lysosomal, regulatory), with VGF highlighted separately. Bar color encodes −log_10_FDR, reflecting enrichment significance. Background values were computed using the same enrichment method as for NPTX2, providing direct reference points for evaluating the relative magnitude and specificity of NPTX2 enrichment across molecular functions. (B) Barplot of the top GO:MF terms enriched among genes correlated with NPTX2 protein, displayed as in (A) with bold bar = NPTX2 protein and thin bar = NPTX2 mRNA. (C–E) Enrichment plots for RNA-derived GO:MF categories—unfolded protein binding, ubiquitin-like protein ligase binding, and translation regulator activity—showing the ranked gene list (x-axis), vertical ticks marking set members, and the running-sum enrichment score (ES). The NES and leading-edge contributors are annotated. (F) Enrichment plot for GO:MF transcription regulator activity based on NPTX2 protein correlations.

In the Molecular Function (MF) analysis, three categories were uniquely enriched in correlation with NPTX2-RNA panel (Fig. 6A). These were unfolded protein binding, ubiquitin-like protein ligase binding, and translation regulator activity. In the protein panel (Fig. 6B), the only unique enrichment was transcription regulator activity. Other MF terms such as transporter activity, neurotransmitter receptor activity, and gated channel activity were observed but overlapped strongly with background synaptic proteins and were not specific to NPTX2.

The enrichment curve for unfolded protein binding (Fig. 6C) had an NES of 12.278. Positive contributors were DNAJA4, HYOU1, PFDN2, HSP90AB1, and CCT8, and the negative contributor was HSPB6. In controls, these genes correlated positively with NPTX2, but in AD the correlations were reduced and HSPB6 became more negatively correlated.

For ubiquitin-like protein ligase binding (Fig. 6D), NES was 12.235. Positive contributors were EGR2, TPI1, RTN4, HSP90AB1, and ATP6V0C, while the five negative contributors were PAX6, EGFR, PER3, RHOBTB3, and TRIOBP. In controls, these genes correlated positively with NPTX2, while in AD correlations were reduced or shifted negatively.

Translation regulator activity (Fig. 6E) had an NES of 9.855. The five positive contributors were AARS1, EIF5A, EIF4A3, EIF1B, and EIF2S2, and the negative contributor was EIF4EBP2. In controls, positive correlations were observed between NPTX2 and translation factors, but in AD these correlations weakened, and EIF4EBP2 shifted to negative correlation.

At the protein level, transcription regulator activity (Fig. 6F) showed enrichment with a biphasic profile (NES = 11.883). Positive contributors were NEUROD6, ATOH7, PEG3, CRYM, and SUB1, and negative contributors were SMAD5, ZIC4, JDP2, ZHX3, and FOXJ1. In controls, positive correlations between NPTX2 protein and neuronal transcriptional activators were maintained in AD, while negative correlations with repressors increased in strength.

## DISCUSSION

NPTX2 is a synaptic gene consistently reduced in Alzheimer’s disease (AD). Integrating bulk RNA-seq and targeted proteomics across four human cohorts, we confirm its down-regulation and reveal gene-specific patterns of correlation in control brain and disrupted correlations (ie. Decorrelation) in AD. Decorrelations in AD reflect both synaptic loss and broader reorganization of neuronal and metabolic networks.

Across the cohort, NPTX2 RNA and protein levels were moderately correlated, consistent with common divergence between mRNA and protein abundance attributable to translational and post-translational regulation [24]. Several genes exhibited distinct associations with NPTX2 (Fig. 2). BDNF showed strong correlation with both NPTX2 RNA and protein, consistent with its role as a direct transcriptional driver of NPTX2 and mediator of BDNF-dependent plasticity [26]. VGF likewise correlated across modalities, in agreement with prior findings linking VGF and NPTX2 levels in CSF and brain and their co-membership in an activity-regulated synaptic module [27]. SCG2 and SST maintained RNA–protein coupling with NPTX2; both are activity-dependent neuronal transcripts downregulated in AD. Along with BDNF, they constitute the co-regulated ‘VGF network hub’ supporting excitatory–inhibitory balance. SCG2 is notably induced by PV interneuron activity, whereas SST marks somatostatin interneurons within the fast-spiking inhibitory circuit [28–30]. In contrast, immediate-early transcripts DUSP4 and EGR4, although induced by BDNF or synaptic activity, displayed weak or absent RNA correspondence with NPTX2 protein, consistent with their transient expression compared with the slower accumulation of NPTX2 protein [3, 31–33]. SERTM1 showed modest correlation with both NPTX2 RNA and protein, confirming prior transcriptomic signals of AD-related dysregulation and identifying it as a novel candidate gene [34].

Within-group analyses revealed how NPTX2 partnerships shift in AD (Fig. 3). The attenuation of NPTX2–VGF correlation at the RNA level was observed, while their RNA–protein relationship remained largely stable, paralleling prior CSF results showing persistence but weakening of this association [27]. BDNF exhibited transcript-level decorrelation with NPTX2 while maintaining moderate RNA–protein correlation. Among inhibitory-circuit genes, SST and SCG2 diverged: SST showed transcript-level decorrelation with preserved RNA–protein correlation, whereas SCG2 displayed broader loss across layers [28, 35, 36]. SERTM1 behaved similarly to SST, suggesting selective preservation of protein-level coordination despite transcriptional disruption. Immediate-early genes diverged most sharply at the protein layer: DUSP4 retained transcript correlation but lost association with NPTX2 protein, and EGR4 lost both transcript and protein correlation [34]. Collectively, the data confirms reduced coherence of NPTX2 with VGF and reveals additional, gene-specific decorrelations involving BDNF, SST, SCG2, SERTM1, DUSP4, and EGR4, signifying reorganization rather than uniform collapse of the NPTX2-centered network.

Principal component analysis (PCA) and correlation heatmaps further clarified NPTX2’s network placement (Fig. 4). Both RNA and protein NPTX2 are localized along PC1, a neuronal/energetic axis enriched for synaptic and mitochondrial genes (e.g., SYT1, GRIA4, HOMER1, RAB5A, VDAC1). NPTX2 thus anchors a compact “plasticity” module that fluctuates independently of stress and lysosomal programs. Several genes showed noncanonical module membership. HDAC2 grouped with neuronal vesicle/mitochondrial genes rather than the chromatin/stress cluster, consistent with its enrichment in mature neurons and reported elevation in AD cortex [37–40]. Notably, our data show a positive correlation between HDAC2 RNA and synaptic-gene RNA, contrary to reports of elevated HDAC2 in AD when synaptic transcripts are reduced, indicating that cell-type–specific analyses are needed to determine whether this relationship reflects differences in neuronal versus glial expression. HDAC8 and HDAC9 aligned with RELN, bridging structural and regulatory functions [41–43]. SQSTM1 (p62) and HNRNPA2B1 clustered within the pathology/stress module, consistent with their known accumulation in neurofibrillary tangles and tau-associated RNA-binding pathology, respectively [44, 45]. RPS6KA5 (MSK1) is associated with inflammatory signatures, linking neuronal stress to microglial NF-κB activation [43]. These contextual placements refine the molecular landscape in which NPTX2 operates [46].

Cross-modality comparisons showed that synaptic proteins closest to NPTX2—its own protein and VGF—retained partial alignment with RNA-based neuronal modules, whereas trafficking and membrane-associated proteins exhibited weaker or inverse correspondence. Collectively, PCA and correlation structure confirm that NPTX2 resides on a principal neuronal/metabolic axis, defining a cohesive plasticity cluster embedded within the AD-altered network.

To interpret pathway-level organization, we performed gene-set enrichment analysis (GSEA) using Gene Ontology (GO) categories. Within GO: Cellular Component, NPTX2 aligned with expected neuronal compartments but also showed strong associations with proteostasis, translation, and transcriptional regulation (Fig. 5). In the Synapse category, NPTX2 clustered with BDNF, VGF, SST, and EGR3—genes reduced in AD cortex and CSF and critical for excitatory–inhibitory balance and vesicle cycling [47–50]. Negatively associated transcripts (ATP1A2, SLC6A11, ADD3, FAM107A, PSD2) were enriched for astrocytic or endolysosomal functions, consistent with a disease-related shift from neuronal to glial programs [51–55]. Within the Mitochondrion category, NPTX2 co-varied with energy-supporting genes (BDNF, RGS2, SH3BP5, MTCH1, PDK3) and was inversely associated with fatty-acid and acetyl-CoA metabolism genes (ACSS1, ECHDC2, ACOT11, AIFM3, ACSS3), reflecting reweight from oxidative neuronal metabolism toward compensatory lipid pathways [47, 56–60].

At the RNA level, three GO: Molecular Function categories—unfolded-protein binding, ubiquitin-like protein ligase binding, and translation-regulator activity—were enriched among NPTX2-correlated transcripts (Fig. 6). Unfolded-protein binding highlighted chaperones such as DNAJA4, HYOU1, PFDN2, HSP90AB1, and CCT8, whereas HSPB6 was inversely correlated, suggesting that stress-responsive elements diverge from the NPTX2-aligned neuronal program [61–63]. Ubiquitin-like ligase binding genes (EGR2, TPI1, RTN4, HSP90AB1, ATP6V0C) linked NPTX2 to protein quality control and membrane regulation [50, 63–66], while negative contributors (TRIOBP, RHOBTB3, PER3, EGFR, PAX6) emerged as novel candidates with limited prior connection to AD [67, 68]. Translation-regulator activity connected NPTX2 to core components of the translational apparatus (AARS1, EIF5A, EIF4A3, EIF1B, EIF2S2) [69–71], whereas EIF4EBP2, a neuronal translation repressor, was inversely correlated with NPTX2, suggesting a link to mTOR-driven translation in AD [72].

At the protein layer, transcription-regulator activity was uniquely enriched. Positive contributors included neuronal differentiation factors (NEUROD6, ATOH7, PEG3, CRYM, SUB1), many reduced in AD [73–75], while negative contributors—FOXJ1, ZHX3, JDP2, ZIC4, SMAD5—represent stress or developmental repressors whose strengthened inverse associations with NPTX2 protein in AD point to an emerging repressive transcriptional environment [76–79]. In particular, ZHX3, typically downregulated in senescent cells, showed enhanced negative correlation with NPTX2 protein as its abundance declined, nominating it as a novel marker of synaptic repression [80].

Across modalities, a consistent contrast emerged. Transcript-level networks—including synaptic, mitochondrial, proteostasis, and translational genes—lost coordinated alignment with NPTX2 in AD, reflecting disintegration of normal neuronal coupling. Proteomic networks, in contrast, retained or even strengthened associations with repressive transcriptional regulators. Rather than a global breakdown, these patterns indicate selective reweighting of the molecular environment surrounding NPTX2: weakening of transcriptional synchrony but reinforcement of inhibitory and stress-linked control at the protein level.

Together, these findings place NPTX2 at the nexus of neuronal integrity, integrating activity-regulated transcription, synaptic homeostasis, and proteostasis balance. In AD, this coordinated system fragments: RNA-level synchrony between NPTX2 and other synaptic or metabolic programs collapses, while proteomic signatures shift toward repressive transcriptional states. The emergence of previously understudied negative correlates—TRIOBP, RHOBTB3, PER3, PAX6, ZHX3, ZIC4, and FOXJ1—extends the NPTX2 landscape beyond known synaptic markers and points to new regulatory mechanisms that may constrain plasticity in disease. These results argue that loss of NPTX2 in AD reflects not merely synaptic attrition but a coordinated failure of neuronal maintenance pathways linking transcription, translation, and proteostasis.

## METHODS

### Sample Acquisition

Fresh-frozen cortical punches from the middle temporal gyrus were obtained from four brain banks: Banner Sun Health Research Institute; University of California, Irvine; Johns Hopkins University; and Northwestern University.

### Brain Bank Contributors

#### Banner Sun Health Brain and Body Donation Program (BBDP)

The Brain and Body Donation Program at Banner Sun Health Research Institute is a prospective clinicopathologic cohort initiated in 1987 that recruits mainly from retirement communities in metropolitan Phoenix, Arizona [81, 82]. Enrollees undergo standardized medical, neurologic, and neuropsychological assessments during life, with rapid autopsy at death (median post-mortem interval ~3.0 hours) yielding high-quality frozen tissues, including a median brain RNA Integrity Number of 8.9 [81–84]. Whole-body donation has been available since 2005, and neuropathologic diagnoses are rendered by licensed pathologists using contemporary consensus criteria [81].

#### 90+ Study

The 90+ Study is a population-based longitudinal cohort of adults aged 90 years and older, drawn primarily from surviving members of the Leisure World Cohort in Laguna Woods, California [85]. Participants are evaluated approximately every 6 months with a neurologic examination, a standardized neuropsychological battery, and informant questionnaires; remote phone or mail assessments are used when in-person visits are not feasible [85, 86]. Dementia is diagnosed by study clinicians, and a brain-donation program provides neuropathologic confirmation using CERAD and NIA-Reagan criteria [85]. Additional cohort descriptions and epidemiologic findings on dementia prevalence and incidence in this population have been published [87, 88].

#### Northwestern University SuperAging Study

This longitudinal cohort enrolls community-dwelling adults aged 80 years or older who perform at or above the mean for 50–60-year-olds on Rey Auditory Verbal Learning Test delayed recall and within the average range for their age on non-memory measures; recruitment occurs through the Alzheimer’s Disease Center and community outreach [89, 90]. Participants complete standardized neuropsychological evaluations at baseline and follow-up, with optional neuroimaging and biomarker studies and brain donation for neuropathology [89]. A recent 25-year program overview summarizes recruitment, phenotyping, and longitudinal expansion [91].

#### Johns Hopkins Division of Neuropathology Brain Bank

This autopsy cohort draws brain donations through the Maryland Office of the Chief Medical Examiner and affiliated programs, explicitly including young adults; standardized dissections and region-specific sampling are performed at the Johns Hopkins Division of Neuropathology [92]. Tissues consisted of fresh-frozen punches of the left middle temporal gyrus. For each case, detailed medical and toxicology histories are abstracted, and tissue is processed for uniform histology, with genotyping and molecular assays performed when applicable [92]. The cohort has been used to characterize preclinical Alzheimer-type pathology in individuals aged 40–50, informing agestratified analyses of early disease biology [92].

### RNA-seq Workflow

FASTQ files were analyzed using the Nextflow RNA-seq pipeline, including trimming with Trim Galore, alignment with STAR21, and quantification of counts at the gene level with SALMON. Quality control was conducted using MultiQC and Qualimap22. Samples were included if they had all covariates, at least 20 million sequencing reads, and if 80% of those reads were uniquely mapped to the human transcriptome. Raw counts were imported from DESeq223 and, for quality control purposes, transformed using the vst method. We removed genes located on the sex chromosomes and conducted Principal Component Analysis (PCA) to identify outlier samples, defined as those that were above ± 3 standard deviations from the average of at least one of the two top PCs.

After quality control, a total of 575 samples were included in the analysis. The control group comprised 180 cognitively normal individuals aged over 50 years. The mild cognitive impairment (MCI) group included 83 individuals diagnosed with MCI and exhibiting Alzheimer’s disease (AD) neuropathological changes. The AD group consisted of 218 individuals with a clinical diagnosis of dementia and AD high pathology, defined as Braak stage III–VI and CERAD rated as “moderate” or “high”. An additional 94 samples were analyzed and categorized as “others”. This group included individuals with a clinical diagnosis of dementia but lacking AD pathology, as well as cognitively normal individuals younger than 50 years.

### PRM-MS Workflow

Human brain tissue samples were homogenized by sonication in a lysis buffer containing 8 M urea, 50 mM triethylammonium bicarbonate (TEAB). The homogenate was centrifuged at high speed, and the resulting supernatant was collected for subsequent processing. The protein concentration of the brain lysate was determined using a bicinchoninic acid (BCA) assay. For each sample, a predetermined amount of protein was aliquoted and supplemented with synthetic isotopically-labeled peptide standards (SpikeTides^™^ L, JPT Peptide Technologies). These standards, featuring ^13^C- and ^15^N-labeled lysine and arginine residues, served as internal references for precise parallel reaction monitoring-mass spectrometry (PRM-MS) quantification. Reduction and alkylation of cysteine residues were conducted by adding tris(2-carboxyethyl) phosphine (TCEP) to a final concentration of 20 mM and incubating for 1 hour at room temperature. This was followed by alkylation with 80 mM chloroacetamide (CAA) for 30 minutes in the dark. A sequential enzymatic digestion strategy was employed for efficient protein cleavage. First, Lys-C endopeptidase (Wako Chemicals) was added at a 1:50 (w/w) enzyme-to-protein ratio and incubated for 3 hours at 37°C. The sample was then diluted with 50 mM TEAB to reduce the urea concentration to 2 M. Sequencing-grade modified trypsin (Promega) was added at a 1:50 (w/w) ratio, and digestion proceeded overnight at 37°C. The digestion was quenched by acidification with trifluoroacetic acid (TFA). The resulting peptide mixture was desalted using C18 StageTips (3M Empore^™^). The eluted peptides were dried in a SpeedVac vacuum concentrator (Thermo Fisher Scientific) and stored at −80°C until LC-MS/MS analysis.

Liquid chromatography-tandem mass spectrometry (LC-MS/MS) analyses were conducted based on established principles of targeted proteomics [93], with specific optimizations for the current application. An Orbitrap Fusion Lumos Tribrid mass spectrometer (Thermo Fisher Scientific), correlated to an Ultimate 3000 RSLCnano liquid chromatography system (Thermo Fisher Scientific), was used for all experiments. For chromatographic separation, tryptic peptides were first loaded onto a C18 trap column (Acclaim PepMap100, 100 μm × 2 cm, 5 μm; Thermo Fisher Scientific) at a flow rate of 5 μL/min.

Analytical separation was then achieved on an EASY-Spray analytical column (50 cm × 75 μm, 2 μm C18 particles; Thermo Fisher Scientific) using a linear gradient from 6% to 28% solvent B (0.1% formic acid in 95% acetonitrile) over 55 minutes at a constant flow rate of 250 nL/min. The ion source was operated at a spray voltage of 2.0 kV. Data acquisition was performed in a targeted MS2 mode. A full MS1 scan (*m/z* 300–1600) was acquired in the Orbitrap at a resolution of 120,000 (at *m/z* 200) for profiling. Subsequently, precursor ions corresponding to the target peptides and their isotopically labeled standards were selectively fragmented via higher-energy collisional dissociation (HCD), and the resulting MS2 spectra were recorded in the Orbitrap at a resolution of 30,000. The automatic gain control (AGC) targets were set to 500,000 ions for MS1 and 100,000 ions for MS2. All raw data were processed and quantitatively analyzed using the Skyline software environment [94]. Peak areas from the extracted ion chromatograms of the target fragment ions were used to calculate the relative abundance of each endogenous peptide against its corresponding heavy internal standard for precise quantification.

To derive protein-level abundance values from peptide-level abundance values, a multi-step normalization and aggregation strategy was implemented. Initially, the raw peak area for each individual peptide was normalized to its median value across the entire sample set. Subsequently, these normalized values underwent a log_2_ transformation. Finally, for every target protein, the transformed values from all associated peptides were averaged to produce a single, consolidated protein abundance estimate.

### Integrated Multi-Omics Analysis

For the PRM-MS analysis, we targeted proteins representing major pathways of interest, encompassing synaptic, trafficking, lysosomal/autophagy, and regulatory or modulatory functions. The synaptic panel included SYT1, RIMS1, HOMER1, GRIN2B, GRIA4, NPTX1, NPTX2, and NPTXR, covering presynaptic release machinery and postsynaptic scaffolds and receptors. Trafficking factors RAB5A and RAB11A were incorporated to reflect endosomal GTPase activity, while lysosomal and autophagic components ATP6V1H, SQSTM1, LAMP1, and LAMP2 captured degradation and clearance pathways. The regulatory/modulator set—VDAC1, DPP6, RHEB, PHF24 (KIAA1045), HNRNPA2B1, and VGF— represented genes involved in mitochondrial homeostasis, excitability, mTOR signaling, RNA processing, and activity-dependent plasticity. In addition to this proteomic panel, exploratory RNA-only candidates were analyzed to capture molecular programs without proteomic coverage, including complement components (C1QA, C1QB, C1QC) for microglial synaptic pruning, chromatin and stress regulators (HDAC1, HDAC4–10, NFE2L2) for transcriptional repression and oxidative-stress defense, synaptic and circuit markers (PVALB, RELN, RIMS2) reflecting interneuron and dendritic function, and stress-responsive signaling genes (RPS6KA5 [MSK1], YWHAG [14-3-3γ]) linked to kinase and adaptor mechanisms. Together, these sets provided complementary coverage of neuronal, glial, and regulatory systems relevant to synaptic and metabolic integrity in Alzheimer’s disease.

Group-level comparisons of NPTX2 RNA and NPTX2 protein across diagnostic categories (Control, MCI, AD) were performed using Wilcoxon rank-sum tests with Benjamini–Hochberg (BH) correction for three pairwise contrasts, and adjusted p-values were annotated on boxplots. For correlation analyses, gene-wise Pearson correlations were computed with NPTX2 RNA or protein using pairwise complete observations, discarding results with fewer than three valid samples or zero variance. P-values were derived via Fisher z-transformation and adjusted using BH. Volcano plots displayed −log_10_(FDR) versus correlation coefficient (r), with thresholds of |r| ≥ 0.50 (moderate), |r| ≥ 0.30 (weak), and otherwise considered nonsignificant. Gene–NPTX2 scatterplots included regression lines and 95 % confidence ellipses for Control and AD groups, and group separation was quantified using pooled-covariance Mahalanobis distance. Correlation matrices were then computed across selected RNA genes, proteomic variables, and metadata; hierarchical clustering employed average linkage and distance = 1 – r, generating modules annotated by biological content such as synaptic machinery, mitochondrial/vesicle transport, lysosomal/autophagic factors, chromatin/stress regulators, and AD pathology metrics. Principal component analysis (PCA) was applied to variance-stabilized RNA expression values, and PC1 and PC2 loadings were used as quantitative phenotypes for enrichment and visualization. PC1 primarily aligned with synaptic and mitochondrial modules, interpreted as a neuronal integrity axis, whereas PC2 aligned with glial, lysosomal, and myelin-associated genes, representing a neuroinflammatory or white-matter axis.

Functional enrichment analyses followed a continuous-phenotype GSEA framework. Genes were ranked by Pearson correlation with NPTX2 RNA, NPTX2 protein, or PCA loadings (PC1, PC2), and enrichment scores (ES) were computed as mean rank within set minus mean rank overall. Significance was assessed against 5,000 phenotype-permuted null distributions to generate normalized enrichment scores (NES = [ES – mean_null_]/sd_null_); two-sided p-values were obtained from the standard-normal approximation, with FDR controlled by BH. Leading-edge subsets comprising the top five positive and negative contributors (Pos5/Neg5) were extracted per term. Enrichment used Gene Ontology (BP, MF, CC), KEGG, Hallmark, Transcription Factor Target (TFT), and curated disease sets from MSigDB v2025, with obsolete entries pruned. To distinguish NPTX2-specific enrichments from general synaptic or metabolic co-expression, background gene groups representing synaptic, trafficking, lysosomal, and regulatory modules were used for comparison. A pathway was considered unique if |NES_nptx2_| exceeded all background medians by ≥ 1.2-fold. Comparative analyses included (1) NPTX2 vs VGF, visualized via NES barplots across RNA and protein data; (2) cross-modality RNA vs protein comparisons of NPTX2 NES values; and (3) PCA-based GSEA comparing PC1- and PC2-associated enrichments to interpret functional axes of molecular variation.

Pairwise correlations across all measured variables were recalculated using complete sample counts, with two-sided p-values derived from t-tests on r and log-space FDR correction for numerical stability. Associations with |r| ≥ 0.70 were classified as strong after collapsing symmetric duplicates. All statistical analyses and visualizations were performed in R v4.4.2 under macOS Sequoia 15.1.1. Figures—including boxplots, scatterplots, enrichment barplots, volcano plots, PCA biplots, and correlation heatmaps—were produced using ggplot2 v4.0.0, ggrepel v0.9.6, cowplot v1.2.0, gplots v3.2.0, ggnewscale v0.5.2, and ggpubr v0.6.1, with additional utilities from gridExtra v2.3. Parallelized permutation testing employed future.apply v1.20.0 in multisession mode, reserving one core. Core analytical packages included dplyr v1.1.4, tibble v3.3.0, readr v2.1.5, stringr v1.5.2, igraph v2.1.4, ggraph v2.2.2, car v3.1–3, limma v3.62.2, and fgsea v1.32.4. Random seeds were fixed to ensure reproducibility across runs. A large language model (LLM) was employed solely for stylistic editing and grammar review during manuscript preparation to improve accessibility across disciplines; no analytical content was generated by the model.

### Large Language Model Use

A large language model (LLM) was used during manuscript preparation to check spelling and grammar, improve readability, and broaden the manuscript’s accessibility to scientific disciplines beyond those specializing in the article’s main topics.

## Supplementary Material

1
