# Supplementary material for: Transcriptomic and protein analysis of human cortex reveals genes and pathways linked to NPTX2 disruption in Alzheimer’s disease
Source: bioRxiv. 2025 Oct 20:2025.10.17.683150. Preprint. [Version 1] doi: 10.1101/2025.10.17.683150 (PMC12633229; doi:10.1101/2025.10.17.683150)
Supplement: 1 [file NIHPP2025.10.17.683150V1-supplement-1.pdf]

(which was not certified by peer review) is the author/funder, who has granted bioRxiv a license to display the preprint in perpetuity. It is m
available under aCC-BY 4.0 International license.

Supplemental Table 1: Summary of gene ontology cellular component and molecular function annotations for the eight RNA transcripts with |r| > 0.5 correlation to NPTX2 mRNA.

Gene	Core Brain Function	Cell type
BDNF	Master neurotrophins that drive neuronal survival, dendritic growth, and long-term synaptic plasticity	Broadly neuronal, highest in cortical and hippocampal pyramidal neurons; also in astrocytes under stress
DUSP4	Activity-inducible phosphatase that dampens ERK/MAPK signaling to tune neuronal excitability and gene expression	Predominantly cortical and hippocampal neurons (inducible); low in microglia/astrocytes
VGF	Secretory-granule precursor whose neuropeptides enhance synaptic efficacy	Excitatory and inhibitory neurons of cortex, hippocampus, hypothalamus
EGR4	Immediate-early zinc-finger transcription factor coordinating activity-driven genes for synapse maturation	Activity-responsive excitatory neurons in cortex/hippocampus
SCG2	Secretogranin II that packages neuropeptides and facilitates Ca^{2+}-dependent neurotransmitter release	Widespread neurons (cortex, limbic, endocrine hypothalamus)
SST	Gi/o-coupled SST receptor (SSTR1-5) signaling	GABAergic SST-interneurons in cortex & hippocampus; neuroendocrine cells
SERTM1	Serine-rich transmembrane protein; putative role in vesicle/ER organization	Enriched in cortical and hippocampal neurons
